# Symptom duration is associated with esophageal remodeling in achalasia treated by peroral endoscopic myotomy

**DOI:** 10.1038/s41598-026-62349-x

**Published:** 2026-07-13

**Authors:** Chunyu Yang, Lanqing Ma, Tao Zhang, Gang Yang, Yuping Wu, Rendi Deng, Xiangqian Dong

**Affiliations:** Department of Gastroenterology, The First Affiliated Hospital of Kunming Medical University, Kunming, China

**Keywords:** Achalasia, Peroral endoscopic myotomy, Symptom duration, High-resolution manometry, Barium esophagram, Treatment outcomes, Diseases, Gastroenterology, Medical research

## Abstract

Symptom duration may reflect cumulative disease burden in achalasia, but its relation to objective recovery after peroral endoscopic myotomy (POEM) remains unclear. In this single-center prospective observational cohort, 68 eligible patients treated with POEM were stratified by preoperative symptom duration (< 3 years, 3–10 years, and > 10 years). Clinical success was defined as an Eckardt score ≤ 3 at 3 and 6 months. Secondary outcomes included perioperative safety, persistent symptoms, high-resolution manometry, barium esophagram morphology, and subtype distribution. All patients underwent technically successful POEM without severe adverse events, and all achieved clinical success at both follow-up time points; therefore, the study could describe short-term clinical response but could not identify predictors of clinical failure. Longer symptom duration was associated preoperatively with more prior treatment, lower integrated relaxation pressure and lower esophageal sphincter pressure, more advanced esophageal remodeling, fewer type II cases, and more unclassified cases. Among 58 patients with objective 3-month reassessment, manometric and morphologic parameters improved significantly after POEM, although morphologic recovery was less complete in patients with the longest symptom duration. These findings suggest that symptom duration is better interpreted as a marker of cumulative disease remodeling than as an isolated determinant of short-term symptomatic success after POEM.

**Supplementary Information:**

The online version contains supplementary material available at https://doi.org/10.1038/s41598-026-62349-x.

## Introduction

Achalasia is an uncommon primary esophageal motility disorder characterized by impaired relaxation of the lower esophageal sphincter (LES) and absent or abnormal peristalsis of the esophageal body^1^. Patients typically present with dysphagia, regurgitation, chest pain, and weight loss. Since its first description in 2010 by Inoue and colleagues^2^, peroral endoscopic myotomy (POEM) has become an established first-line treatment because of its minimally invasive nature and durable short- and long-term efficacy^1,3^.

The clinical course of achalasia, however, is heterogeneous. Some patients undergo definitive treatment soon after symptom onset, whereas others live with symptoms for many years before intervention. Symptom duration may therefore serve as an easily obtainable marker of cumulative disease burden. Previous studies have suggested that longer symptom duration and prior therapy may be associated with more advanced esophageal remodeling, greater technical complexity, or differences in postoperative recovery, but the available evidence remains inconsistent^3–5^.

A major limitation of earlier studies is that symptom duration has rarely been evaluated together with objective structural parameters, such as esophageal dilation diameter and angulation, or with postoperative morphologic recovery. In addition, the relationship between symptom duration and Chicago subtype distribution remains insufficiently characterized, particularly in patients whose HRM findings suggest achalasia but do not fit a classic subtype pattern.

We therefore conducted a prospective single-center cohort study to evaluate associations between symptom duration, preoperative disease phenotype, and short-term recovery after POEM for achalasia. We hypothesized that longer symptom duration would be associated with more advanced preoperative morphologic and manometric abnormalities, whereas short-term symptom relief after POEM would remain favorable in this treated cohort.

## Methods

### Study design and patients

This was a single-center prospective observational cohort study. Patients with achalasia who underwent POEM in the Department of Gastroenterology at The First Affiliated Hospital of Kunming Medical University between April 2024 and July 2025 were prospectively enrolled after meeting the predefined eligibility criteria. Recruitment occurred continuously within routine clinical practice. Because a formal screening log of all patients assessed but not enrolled was not maintained, the exact number initially screened and the detailed pre-enrollment exclusion counts could not be reliably reconstructed. To avoid reporting retrospective estimates as observed data, the final analytic cohort comprised 68 patients with documented eligibility and enrollment. The protocol was approved by the institutional ethics committee of The First Affiliated Hospital of Kunming Medical University. All procedures involving human participants were performed in accordance with institutional requirements and the ethical principles of the Declaration of Helsinki, and all participants provided written informed consent.

Eligible patients had symptomatic achalasia diagnosed on the basis of compatible symptoms together with endoscopy, barium esophagram, and HRM. Pre-enrollment exclusion criteria were severe coagulopathy or major comorbidity precluding POEM, refusal of POEM, or absence of essential perioperative records required for baseline analysis. Failure to complete the objective 3-month reassessment after enrollment was not treated as an exclusion criterion; these patients remained in the clinical follow-up analysis. Unclassified achalasia was operationally defined as clinically suspected achalasia with supportive endoscopic and barium esophagram findings but HRM findings that did not meet the classic Chicago type I, II, or III patterns. All 12 such patients had IRP values < 15 mmHg and esophageal body motility that did not satisfy the classic subtype criteria. They were categorized as unclassified achalasia because the combined clinical, endoscopic, and radiographic phenotype supported achalasia^1^, rather than because another motility disorder had been established. Detailed patient-level features are provided in Supplementary Table S6.

### Exposure groups and follow-up assessments

**Fig. 1:**
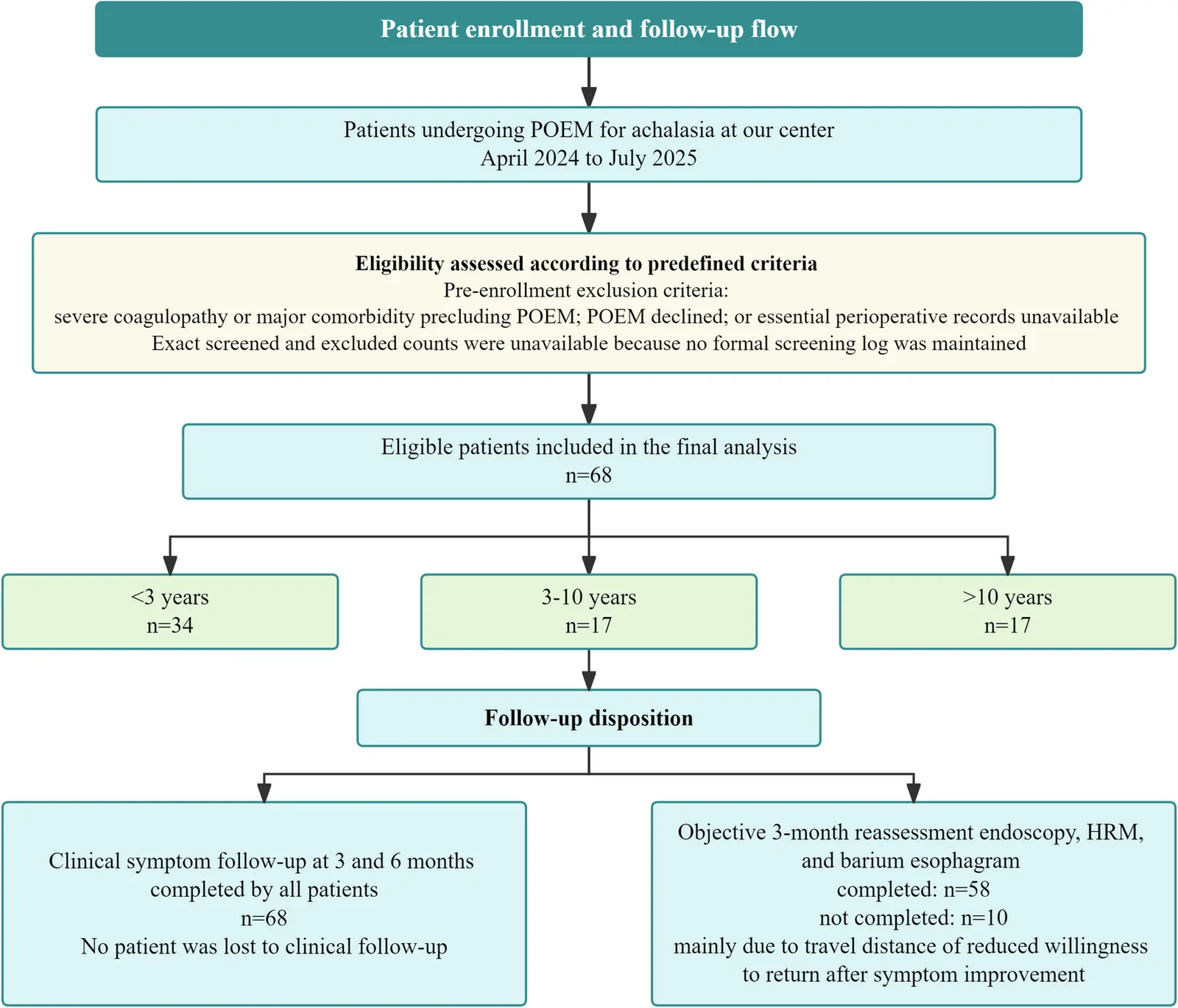
Patient enrollment and follow-up flow diagram.

Symptom duration was defined as the interval from the earliest onset of any symptom ultimately attributed to achalasia—dysphagia, regurgitation, or chest pain—to the date of POEM; alternative causes were excluded during diagnostic evaluation. Symptom duration was determined from patient recall during medical history taking and was cross-checked against outside-hospital or previous medical records when available. If the duration reported by the patient differed from that documented in the medical records, the recorded information was discussed with the patient again and the final symptom duration was determined after reconfirmation. Patients were stratified into three mutually exclusive groups: <3 years (*n* = 34), 3–10 years (*n* = 17), and > 10 years (*n* = 17). All 68 patients completed symptom follow-up at 3 and 6 months after POEM, with key clinical outcomes collected by telephone when in-person follow-up was not performed. In addition, 58 patients underwent repeat endoscopy, HRM, and barium esophagram at 3 months (28 in the < 3-year group, 15 in the 3–10-year group, and 15 in the > 10-year group). The remaining 10 patients completed clinical follow-up but did not complete objective 3-month reassessment, mainly because of long travel distance or reduced willingness to return after satisfactory symptom improvement. The enrollment and follow-up process is summarized in Fig. 1.

Clinical severity was evaluated using the conventional Eckardt score (range 0–12)^6^. Clinical success was defined a priori as a postoperative Eckardt score ≤ 3. Persistent postoperative symptoms were additionally assessed using a modified Eckardt score, in which the frequency category “occasional” is subdivided into more granular intervals; persistent symptoms were defined as symptoms occurring at least monthly or any symptom with a self-reported severity score ≥ 3 on a 5-point Likert scale (0 = none, 1 = mild, 2 = moderate, 3 = severe, and 4 = very severe), where a score ≥ 3 indicated symptoms that substantially interfered with daily dietary habits or activities and impaired quality of life^7^. Postoperative reflux symptoms were screened using the Gastroesophageal Reflux Disease Questionnaire (GerdQ), with a score ≥ 8 considered suggestive of symptom-based reflux rather than an objective diagnosis of gastroesophageal reflux disease^8^.

### Manometry, morphologic assessment, and POEM procedure

HRM was performed preoperatively and again 3 months after POEM. Achalasia subtypes were classified according to Chicago Classification version 4.0^9^. The key manometric variables analyzed were median integrated relaxation pressure (IRP) and lower esophageal sphincter pressure (LESP). Patients with supportive clinical, endoscopic, and barium esophagram evidence of achalasia but non-classic HRM patterns were categorized as unclassified achalasia, as described above. Because only one patient had type III achalasia, this subtype was excluded from multinomial subtype regression analyses to avoid model instability.

**Fig. 2:**
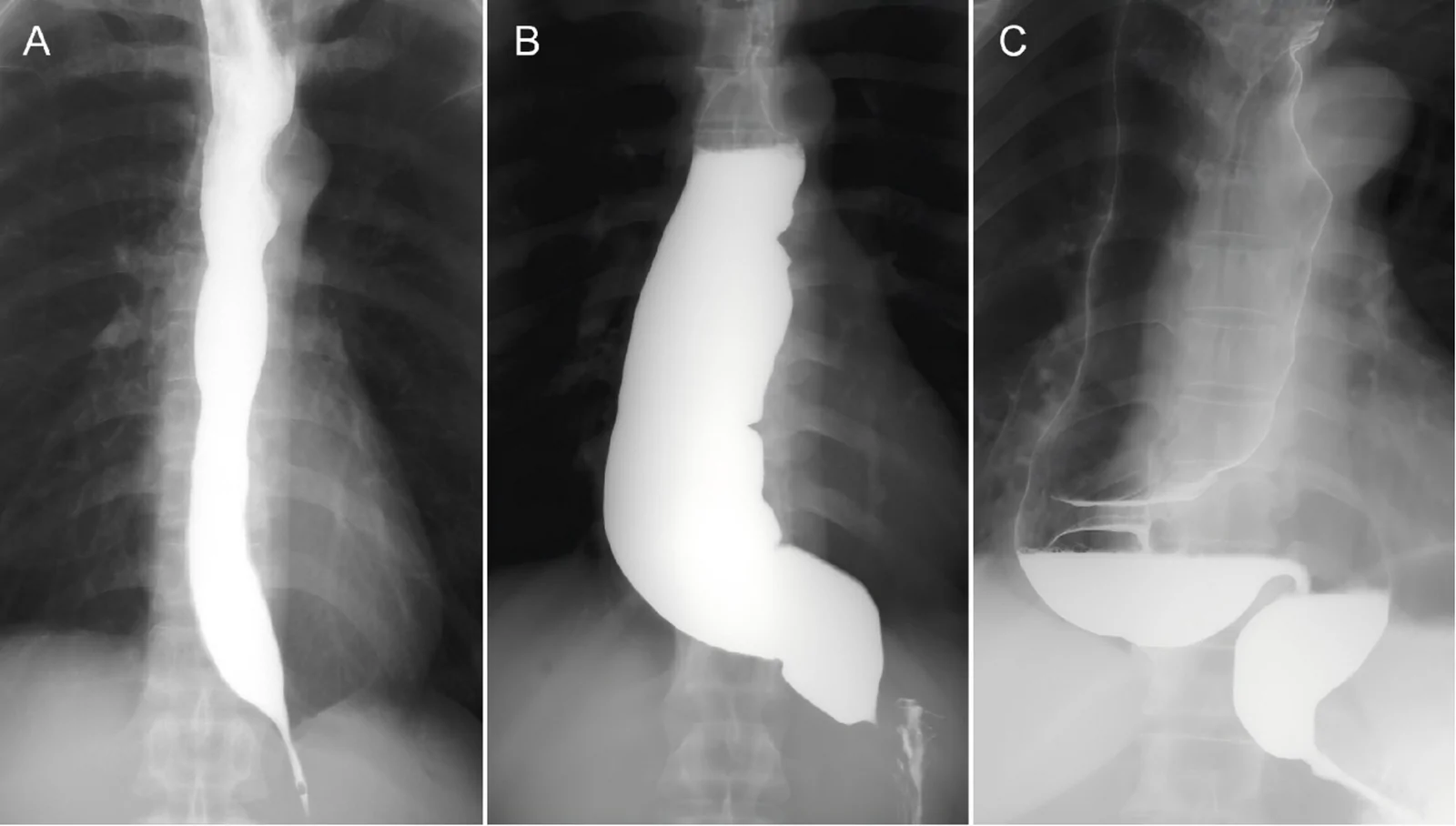
Pre-POEM barium esophagram images. (**A**) Straight-type esophagus with a distal esophageal axis angle ≥ 135° and an esophageal dilation diameter of 37.6 mm. (**B**) Sigmoid-type esophagus with a distal esophageal axis angle of 112° and an esophageal dilation diameter of 58.8 mm. (**C**) Advanced sigmoid-type esophagus with a distal esophageal axis angle of 81° and an esophageal dilation diameter of 69.6 mm.

Barium esophagram was used to measure maximal esophageal dilation diameter, distal narrow diameter, and the angulation of the distal esophageal axis. According to the descriptive rules of the Japan Esophageal Society for the morphologic classification of achalasia, esophageal morphology was categorized as straight type (St, angle ≥ 135°), sigmoid type (Sg, angle 90° to < 135°), or advanced sigmoid type (aSg, angle < 90°)^10^(Fig. 2). These measurements were collected before POEM and at 3-month follow-up to assess morphologic recovery.

All POEM procedures were performed under general anesthesia by a single high-volume endoscopist with experience of more than 300 POEM cases. A posterior approach was used routinely (Supplementary Fig. S1), with individualized modifications in patients with severe tortuosity or prior scar formation. Standard tunnel length was 10–12 cm, whereas short-tunnel POEM (7–8 cm) was used in selected cases with severe tortuosity. Circular myotomy, progressive full-thickness myotomy, or full-thickness myotomy was chosen intraoperatively on the basis of local anatomy and procedural judgment. Technical success was defined as completion of the planned POEM with clip closure of the mucosal entry.

### Endpoints and statistical analysis

The primary endpoint was clinical success at 6 months after POEM, analyzed descriptively because clinical failure did not occur in this cohort. Secondary endpoints included technical success, perioperative adverse events, persistent symptoms, symptom-based reflux defined by a GerdQ score ≥ 8, postoperative HRM parameters, postoperative barium esophagram measurements, and the association between symptom duration and preoperative Chicago subtype.

Continuous variables are reported as mean ± standard deviation or median (interquartile range), as appropriate. Categorical variables are presented as counts and percentages. Between-group comparisons were performed using one-way analysis of variance or the Kruskal–Wallis test for continuous variables and the chi-square test or Fisher exact test for categorical variables, as appropriate. Significant omnibus tests were followed by pairwise independent-samples t tests, Mann–Whitney U tests, or exact tests, as appropriate, with Bonferroni adjustment. For postoperative morphology, the post hoc pairwise comparisons dichotomized morphology as straight versus non-straight and used exact tests with Bonferroni adjustment. Pre- and post-POEM comparisons were performed using paired t tests or Wilcoxon signed-rank tests. Baseline characteristics of patients with and without objective 3-month reassessment were compared using the Mann–Whitney U test or exact tests. Given the limited number of patients and outcome categories, multinomial logistic regression was treated as exploratory; type II achalasia was the reference outcome, type III achalasia was excluded because only one case was observed, symptom duration was coded as < 10 versus > 10 years, and the model was adjusted for prior treatment and baseline esophageal angulation. Spearman correlation coefficients were calculated using exact symptom duration in years. As post hoc sensitivity analyses, subtype distribution and Spearman correlations were repeated in treatment-naive patients. Statistical analyses were performed with SPSS version 26.0. All statistical tests were two-sided, and a P value < 0.05 was considered statistically significant.

## Results

### Baseline characteristics

All 68 enrolled patients underwent POEM. The three symptom-duration groups did not differ significantly in sex distribution, age, body mass index, or baseline Eckardt score (all *P* > 0.05). Prior treatment differed across the three groups (*P* = 0.002) and was most frequent in the > 10-year group (47.1% vs. 11.8% in the 3–10-year group and 5.9% in the < 3-year group) (Table 1). Overall, 12 patients had prior treatment before POEM, including endoscopic balloon dilation in 8 patients, esophageal stent placement in 1 patient, laparoscopic Heller myotomy in 1 patient, combined balloon dilation and stent placement in 1 patient, and prior POEM in 1 patient. Eleven patients had undergone one prior intervention and one patient had undergone three prior interventions; the interval from prior treatment to POEM ranged from 4 months to 20 years, with the exact timing unavailable in one patient (Supplementary Table S1).

**Table 1:** Baseline characteristics by symptom-duration group.

Variable	< 3 years (*n* = 34)	3–10 years (*n* = 17)	> 10 years (*n* = 17)	*P* value
Female/male	16/18	7/10	10/7	0.597
Age, years	38.00 (29.75-52.00)	39.00 (29.00–50.00)	48.00 (42.00–52.00)	0.144
Body mass index, kg/m²	20.59 ± 3.29	20.18 ± 3.17	20.25 ± 3.96	0.904
Prior treatment, n (%)	2 (5.88)	2 (11.76)	8 (47.06)	0.002*
Chicago subtype, n (%)				< 0.001*
Chicago subtype I, n (%)	2 (5.88)	3 (17.65)	5 (29.41)	0.063*
Chicago subtype II, n (%)	28 (82.35)	13 (76.47)	4 (23.53)	< 0.001
Chicago subtype III, n (%)	1 (2.94)	0	0	1.000*
Unclassified achalasia, n (%)	3 (8.82)	1 (5.88)	8 (47.06)	0.002*
Median IRP, mmHg	24.30 (17.10-33.28)	25.20 (16.95–29.90)	12.50 (11.00-16.25)	< 0.001
LESP, mmHg	32.01 ± 12.06	32.97 ± 11.04	16.36 ± 7.73	< 0.001
Esophageal dilation diameter, mm	39.10 (31.03–43.63)	47.50 (39.70-58.65)	41.80 (33.75–54.10)	0.006
Distal narrow diameter, mm	4.80 (3.28–5.80)	4.20 (3.40–4.70)	4.00 (3.45–5.90)	0.354
Esophageal angulation, °	147.00 (141.50–150.00)	133.00 (121.50–142.00)	125.00 (97.50-143.50)	< 0.001
Esophageal morphology, n (%)				< 0.001*
Straight-type morphology, n (%)	30 (88.24)	8 (47.06)	7 (41.18)	< 0.001
Sigmoid-type morphology, n (%)	4 (11.76)	7 (41.18)	8 (47.06)	0.012*
Advanced sigmoid-type morphology, n (%)	0	2 (11.76)	2 (11.76)	0.057*
Baseline Eckardt score	7.24 ± 2.20	8.00 ± 2.55	6.76 ± 2.66	0.323

*Fisher exact test where applicable, IRP integrated relaxation pressure, LESP lower esophageal sphincter pressure. Significant pairwise comparisons were Bonferroni-adjusted. IRP, integrated relaxation pressure; LESP, lower esophageal sphincter pressure.

Preoperative HRM and barium esophagram findings differed substantially across groups. Median IRP and mean LES pressure were lower in the > 10-year group than in both shorter-duration groups after Bonferroni adjustment (all adjusted *P* ≤ 0.005). The overall Chicago subtype distribution differed across groups (*P* < 0.001); the > 10-year group had fewer type II cases and more unclassified cases than either shorter-duration group. Esophageal dilation diameter was greater in the 3–10-year group than in the < 3-year group (adjusted *P* = 0.006), whereas the > 10-year group did not differ significantly from the other two groups after adjustment. Esophageal angulation was more severe in both the 3–10-year and > 10-year groups than in the < 3-year group (both adjusted *P* < 0.001). The overall morphology distribution also differed across groups (*P* < 0.001), with straight-type morphology concentrated in the < 3-year group (Table 1).

### Procedural outcomes and safety

Technical success was achieved in all 68 patients. Procedure duration, intraprocedural blood loss, tunnel length, and length of hospital stay did not differ significantly across the three groups (all *P* > 0.05). Full-thickness myotomy and short-tunnel POEM were used numerically more often in the > 10-year group, but the global comparisons for myotomy type and tunnel length were not statistically significant (*P* = 0.113 and *P* = 0.062, respectively) (Table 2).

**Table 2:** Procedural outcomes and perioperative safety.

Variable	< 3 years (*n* = 34)	3–10 years (*n* = 17)	> 10 years (*n* = 17)	*P* value
Procedure time, min	35.00 (30.00–40.00)	38.00 (35.00-42.50)	39.00 (30.00-45.50)	0.380
Intraprocedural blood loss, mL	1.00 (0.00-1.25)	1.00 (1.00-1.50)	1.00 (0.50–2.50)	0.413
Myotomy type, n (%)				0.113*
Full-thickness myotomy, n (%)	5 (14.71)	5 (29.41)	8 (47.06)	–
Progressive full-thickness myotomy, n (%)	16 (47.06)	7 (41.18)	3 (17.65)	–
Circular myotomy, n (%)	13 (38.24)	5 (29.41)	6 (35.29)	–
Tunnel length, cm	12.00 (12.00–12.00)	12.00 (12.00–12.00)	12.00 (7.75-12.00)	0.062
Postoperative hospital stay, days	5.00 (5.00–6.00)	5.00 (5.00–6.00)	6.00 (5.00–6.00)	0.249
Procedure-related adverse events, n (%)	2 (5.88)	0	1 (5.88)	0.804

*Fisher exact test where applicable.

No severe adverse events were observed. Three postoperative infections resolved with treatment. No patient developed perforation, pneumothorax, subcutaneous emphysema, major gastrointestinal bleeding, or death.

### Clinical follow-up

**Fig. 3:**
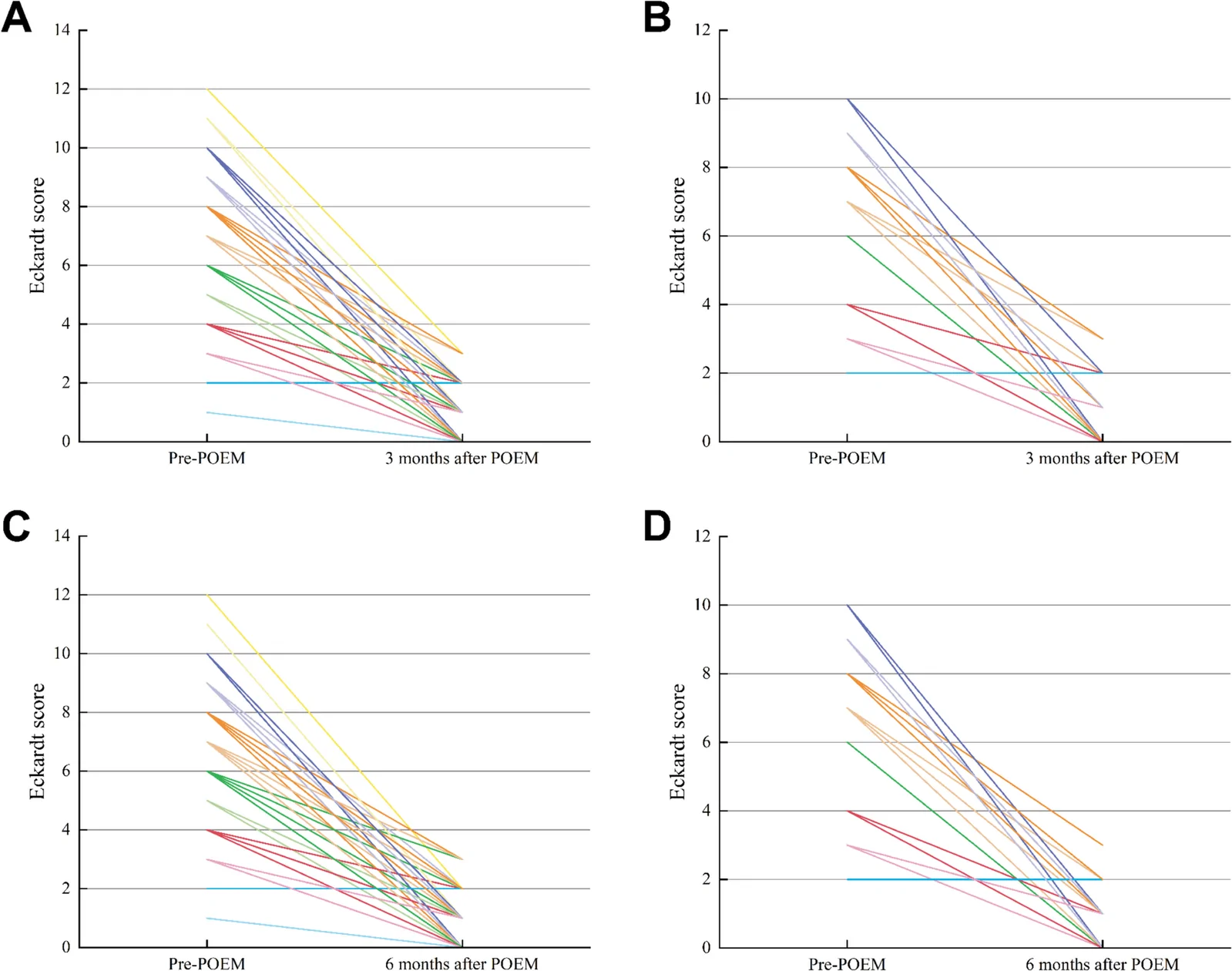
Paired line graphs showing changes in Eckardt scores before and after POEM. (**A**) Overall cohort, baseline versus 3 months. (**B**) Patients with symptom duration > 10 years, baseline versus 3 months. (**C**) Overall cohort, baseline versus 6 months. (**D**) Patients with symptom duration > 10 years, baseline versus 6 months.

At both 3 and 6 months, all patients met the study definition of clinical success, with Eckardt scores ≤ 3 in every case (Fig. 3; Supplementary Table S2). Because no clinical failures occurred, symptom duration could not be evaluated as a predictor of clinical failure. Mean Eckardt scores decreased significantly from baseline in each symptom-duration group at both follow-up time points (all *P* < 0.001) (Supplementary Table S2), but neither the postoperative scores nor the magnitude of score reduction differed significantly across groups (Supplementary Table S3).

Persistent symptoms defined by the modified Eckardt score were more commonly driven by dysphagia than by reflux or chest pain. However, the proportions of patients with persistent dysphagia, reflux, or chest pain did not differ significantly across symptom-duration groups at either 3 or 6 months. The proportion with a GerdQ score ≥ 8 was also comparable across groups (*P* = 0.327) (Supplementary Table S3). Although repeat endoscopy was performed in 58 patients, reflux esophagitis, retained food, and other mucosal findings were not collected using a predefined standardized grading protocol and were therefore not included as analyzable outcomes. Ambulatory pH or impedance–pH monitoring was not performed. Accordingly, a GerdQ score ≥ 8 was interpreted only as symptom-based reflux and not as objective post-POEM GERD.

### Objective reassessment at 3 months

Among the 58 patients who completed objective 3-month reassessment, HRM parameters improved markedly after POEM in every group (Supplementary Table S4). No patient was lost to clinical follow-up at 3 or 6 months. The 10 patients without objective reassessment did not return for repeat endoscopy, HRM, and barium esophagram mainly because of long travel distance or reduced willingness to return after satisfactory symptom improvement. Compared with patients who completed objective reassessment, those without objective reassessment had lower baseline IRP (*P* < 0.001), lower LESP (*P* = 0.014), and a different Chicago subtype distribution (*P* = 0.031), with unclassified achalasia more frequent among non-completers (50.0% vs. 12.1%). Age, BMI, symptom duration, baseline Eckardt score, esophageal dilation diameter, distal narrow diameter, esophageal angulation, sex, prior treatment, and symptom-duration group did not differ significantly (Supplementary Table S5). Both IRP and LES pressure decreased significantly from baseline in each symptom-duration group and in the overall cohort (all *P* < 0.001). After POEM, the < 3-year group retained a higher IRP than the other two groups (*P* = 0.003), whereas the > 10-year group retained the lowest LES pressure (*P* = 0.018) (Table 3).

**Table 3:** Objective 3-month follow-up findings.

Variable	< 3 years (*n* = 28)	3–10 years (*n* = 15)	> 10 years (*n* = 15)	*P* value
Post-POEM IRP, mmHg	9.40 (7.60-10.25)	7.30 (5.70–7.80)	7.30 (4.70–8.20)	0.003
Post-POEM LESP, mmHg	12.00 (9.08–15.90)	12.10 (9.90–14.10)	8.90 (6.60–11.00)	0.018
Esophageal dilation diameter, mm	25.50 (17.87–32.80)	22.80 (20.82-29.00)	26.20 (21.92-37.00)	0.362
Distal narrow diameter, mm	8.15 (7.53–9.15)	8.00 (7.00–9.00)	8.00 (7.20-9.00)	0.838
Esophageal angulation, °	148.00 (145.00-150.00)	143.00 (140.00-146.00)	140.00 (115.00-146.00)	0.002
Esophageal morphology, n (%)				0.040
Straight-type morphology, n (%)	26 (92.86)	13 (86.67)	9 (60.00)	0.026*
Sigmoid-type morphology, n (%)	2 (7.14)	2 (13.33)	4 (26.67)	0.257*
Advanced sigmoid-type morphology, n (%)	0	0	2 (13.33)	0.127*

*Fisher exact test where applicable, IRP integrated relaxation pressure, LESP lower esophageal sphincter pressure. Post hoc exact tests dichotomizing morphology as straight versus non-straight showed a significant difference only between the < 3-year and > 10-year groups after Bonferroni adjustment (adjusted *P* = 0.043).

**Fig. 4:**
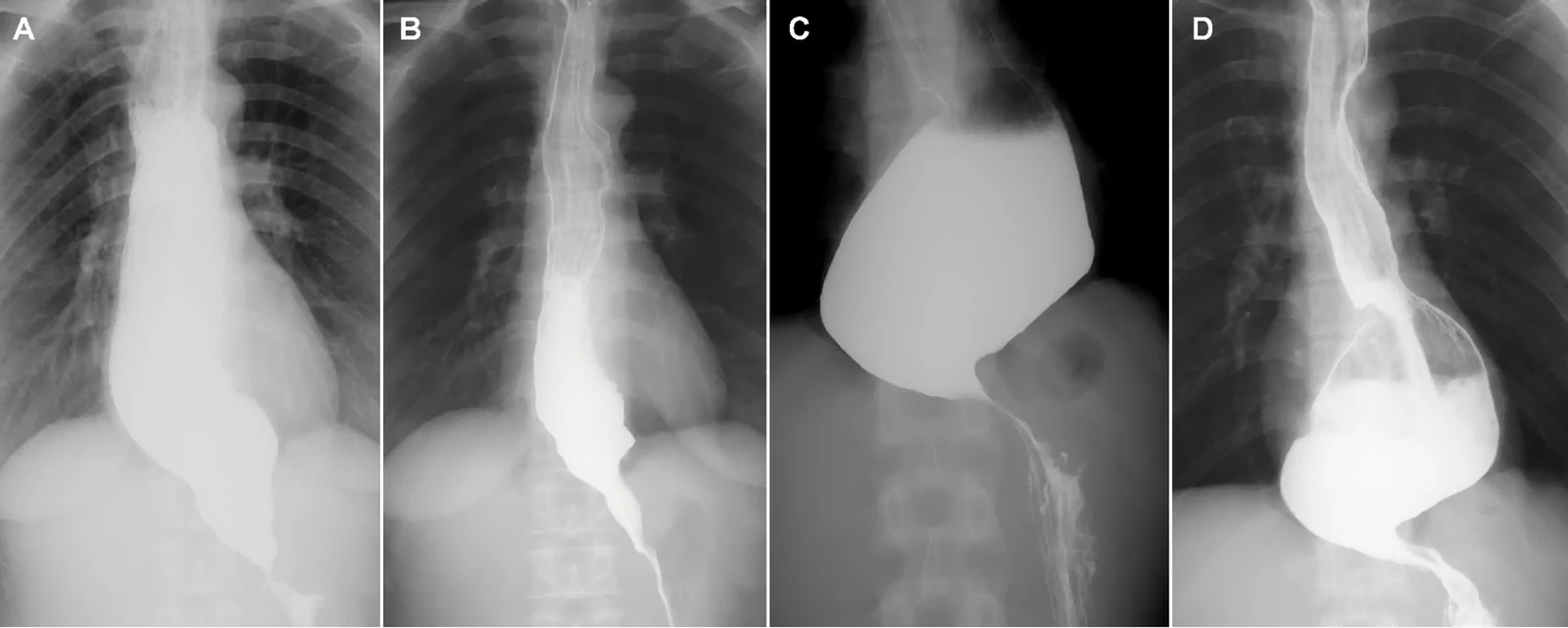
Representative pre- and post-POEM barium esophagram images in patients with different symptom durations. (**A**, **B**) A patient in the 3–10-year group. (**A**) Pre-POEM esophagram showing sigmoid-type esophagus (125°), maximal esophageal dilation diameter of 52.2 mm, and distal narrow diameter of 3.2 mm. (**B**) Post-POEM esophagram showing improvement in esophageal angulation to 141° (straight type), reduction in esophageal dilation diameter to 37.0 mm, smooth contrast passage across the esophagogastric junction, and satisfactory cardia opening. (**C**, **D**) A patient with a 20-year symptom duration. (**C**) Pre-POEM image showing advanced sigmoid morphology. (**D**) Post-POEM image showing persistent advanced sigmoid morphology after POEM.

Barium esophagram measurements also improved significantly after POEM. Esophageal dilation diameter, distal narrow diameter, and esophageal angulation all showed significant pre- to post-procedure improvement in each group and in the overall cohort (all *P* < 0.001) (Supplementary Table S4). Nevertheless, morphologic recovery was less complete in patients with the longest symptom duration. At 3 months, straight-type morphology was present in 92.9% of the < 3-year group, 86.7% of the 3–10-year group, and 60.0% of the > 10-year group. The global morphology distribution differed across groups (*P* = 0.040). In a post hoc analysis that dichotomized morphology as straight versus non-straight, only the comparison between the < 3-year and > 10-year groups remained significant after Bonferroni adjustment (adjusted *P* = 0.043) (Table 3). Figure 4 shows representative pre- and post-POEM barium esophagrams from a patient in the 3–10-year group and from a patient with a 20-year symptom duration in the > 10-year group. In the latter case, advanced sigmoid morphology persisted after POEM.

### Symptom duration, subtype distribution, and correlations

To evaluate the relationship between symptom duration and preoperative Chicago subtype, patients were dichotomized as < 10 versus > 10 years for an exploratory multinomial logistic regression, with type II achalasia as the reference category and adjustment for prior treatment and baseline angulation. Symptom duration < 10 years was associated with lower odds of unclassified rather than type II achalasia (adjusted OR 0.069, 95% CI 0.012–0.400; *P* = 0.003). A trend toward lower odds of type I rather than type II achalasia was also observed for symptom duration < 10 years (adjusted OR 0.169, 95% CI 0.028–1.023; *P* = 0.053) (Table 4). Among the 12 patients with unclassified achalasia, 5 had undergone prior treatment and 7 were treatment-naive. In sensitivity analyses restricted to treatment-naive patients (*n* = 56), the overall subtype distribution remained different across symptom-duration groups (*P* = 0.028). Unclassified achalasia was present in 4 of 9 patients (44.4%) in the > 10-year group compared with 3 of 32 patients (9.4%) in the < 3-year group and 0 of 15 patients in the 3–10-year group; type II achalasia was present in 4 of 9 patients (44.4%) in the > 10-year group compared with 26 of 32 patients (81.3%) and 12 of 15 patients (80.0%) in the < 3-year and 3–10-year groups, respectively (Supplementary Table S7). In treatment-naive patients, symptom duration remained correlated with greater esophageal dilation diameter (rho = 0.396, *P* = 0.003) and more severe esophageal angulation (rho=-0.572, *P* < 0.001), whereas correlations with IRP (rho=-0.129, *P* = 0.345) and LESP (rho=-0.200, *P* = 0.140) were attenuated and no longer statistically significant (Supplementary Table S8).

**Table 4:** Symptom duration, subtype distribution, and correlations.

Analysis	Estimate	95% CI/rho	*P* value
Multinomial logistic regression: <10 years vs. > 10 years, unclassified vs. type II achalasia	OR 0.069	0.012-0.400	0.003
Multinomial logistic regression: <10 years vs. > 10 years, type I vs. type II achalasia	OR 0.169	0.028–1.023	0.053
Spearman correlation: symptom duration vs. IRP	rho − 0.363		0.002
Spearman correlation: symptom duration vs. LESP	rho − 0.403		0.001
Spearman correlation: symptom duration vs. esophageal dilation diameter	rho 0.373		0.002
Spearman correlation: symptom duration vs. esophageal angulation	rho − 0.602		< 0.001

Regression model adjusted for prior treatment and baseline esophageal angulation, IRP integrated relaxation pressure, LESP lower esophageal sphincter pressure.

Spearman analysis showed that symptom duration correlated negatively with IRP (rho=-0.363, *P* = 0.002), LES pressure (rho=-0.403, *P* = 0.001), and esophageal angulation (rho=-0.602, *P* < 0.001), and positively with esophageal dilation diameter (rho = 0.373, *P* = 0.002) (Table 4).

## Discussion

This prospective cohort study yielded three principal findings. First, short-term clinical success after POEM was observed in all patients in this cohort; because no clinical failures occurred, the study cannot determine whether symptom duration predicts success or failure after POEM. Second, longer symptom duration was associated with more advanced preoperative disease, reflected by lower IRP and LES pressure, more severe morphologic distortion, fewer type II cases, and more unclassified cases. Third, although objective morphologic parameters improved after POEM in all groups, postoperative morphologic recovery was less complete in patients whose symptoms had lasted at least 10 years.

Our findings help reconcile the apparently inconsistent literature regarding symptom duration and POEM recovery. Achalasia is often diagnosed only after a substantial delay, and prolonged symptom duration is therefore common in clinical practice^11^. Recent studies have reached differing conclusions regarding the relationship between preoperative symptom duration and outcomes after POEM. Benson et al. reported no significant association between preoperative symptom duration and outcomes after POEM^5^, whereas Sun et al. suggested that long symptom duration may be associated with greater technical complexity and less favorable recovery^12^. Our data support a more cautious interpretation: symptom duration appears to function less as an isolated determinant of short-term symptomatic success and more as a clinical marker of cumulative disease remodeling.

The morphologic findings are particularly important. Chronic functional obstruction at the esophagogastric junction is expected to drive progressive esophageal dilatation, angulation, and transition from a straight to a sigmoid configuration. Prior studies of sigmoid-type achalasia have shown that POEM can still achieve high technical and clinical success rates while improving esophageal angulation and reducing stasis^13–16^. At the same time, those studies suggest that reversibility is incomplete once advanced structural remodeling has developed. In the present study, esophageal dilation diameter, distal narrow diameter, and angulation all improved significantly after POEM, yet residual angulation remained greater and straight-type morphology remained less common in the > 10-year group. These findings support the view that long-standing achalasia may enter a stage of structural remodeling that is only partially reversible even after adequate relief of esophagogastric junction outflow obstruction.

The manometric findings should be interpreted cautiously. In this study, symptom duration was negatively correlated with both IRP and LES pressure, indicating that longer symptom duration was associated with lower IRP and LES values. Previous studies have likewise shown that patients with prior treatment or sigmoid-type esophagus often have lower IRP^13,17^, and that an IRP approaching the normal range may coexist with more advanced esophageal dilation and angulation^18^. These findings suggest that lower IRP and LES pressure in patients with prolonged symptoms should not be interpreted as evidence of milder disease. They may instead reflect structural remodeling or prior therapeutic effects. The higher proportion of unclassified achalasia in the > 10-year group and the adjusted exploratory association between symptom duration and subtype distribution further suggest that prolonged symptoms may be accompanied by a less classic HRM phenotype. This interpretation is consistent with reports that manometric findings do not necessarily mirror symptom severity or structural disease burden^19–21^.

Another clinically important observation is that baseline Eckardt scores were similar across groups despite substantial differences in morphologic and manometric severity. This mismatch indicates that patient-reported symptom severity is not equivalent to cumulative disease burden. Long-term dietary adaptation, altered eating behavior, and progressive habituation to symptoms may all contribute. More refined patient-reported outcome instruments, such as the modified Eckardt score and the Achalasia Patient-Reported Outcomes Questionnaire, may better capture residual symptom burden than the conventional Eckardt score alone^7,22^.

Importantly, our findings do not imply that long symptom duration is a contraindication to POEM. All procedures were completed successfully, no severe adverse events occurred, and all patients achieved short-term clinical success. These favorable short-term outcomes may reflect individualized intraoperative strategy and the experience of the treating endoscopist, but the present study did not directly compare operator experience or test whether experience offset technical difficulty. For patients with marked tortuosity or prior scarring, modifications in tunnel entry site, tunnel length, and myotomy depth may help complete the procedure safely in experienced hands^16,17^.

Technical adaptation may have contributed to the preserved short-term symptom response in the long-duration group. In this cohort, the posterior esophageal wall was used as the primary tunnel route in most cases, and shorter tunnels or modified entry sites were selected when severe distal angulation made a standard tunnel less suitable. Likewise, in patients with prior POEM or other interventions and localized scar formation or fibrotic adhesion, the tunnel was preferentially created through a less fibrotic plane, such as the anterior wall, to reduce technical difficulty and improve safety. Full-thickness myotomy was used more frequently in the > 10-year group, probably reflecting muscular hypertrophy, fibrosis, and anatomic complexity. However, these procedural choices were made intraoperatively and were not tested in a comparative design. Therefore, they should be interpreted as descriptive technical observations rather than evidence that a specific strategy or operator experience causally offset the difficulty of long-standing disease.

These findings have important clinical implications. In patients with symptom duration > 10 years, clinicians may anticipate more advanced morphologic remodeling and a greater likelihood of non-classic manometric features before intervention. After POEM, symptomatic improvement should not be interpreted as complete reversal of the underlying structural abnormalities. Residual esophageal tortuosity and impaired clearance may persist despite adequate relief of esophagogastric junction outflow obstruction. A previous retrospective study reported that esophageal cancer may still occur during follow-up after POEM, with a higher observed incidence in patients with sigmoid-type achalasia^15^. Accordingly, follow-up assessment in patients with long-standing disease may consider residual morphology, esophageal clearance, and mucosal findings in addition to symptom response. However, the present 6-month observational study did not evaluate esophageal cancer risk or the effectiveness or optimal interval of endoscopic surveillance.

Several limitations should be acknowledged. This was a single-center study with a relatively small sample size, which limited subgroup and regression analyses. Because all patients achieved clinical success at 3 and 6 months, predictors of clinical failure could not be evaluated. The absence of a formal screening log may limit assessment of selection bias. Symptom duration was based partly on patient recall, and recall bias remains possible. Although all patients completed clinical follow-up, postoperative HRM and barium esophagram were available in only 58 patients, which may have introduced attrition bias. Post-POEM reflux was assessed using the symptom-based GerdQ, without standardized collection and grading of endoscopic reflux findings or ambulatory reflux monitoring. Finally, the short follow-up period and single-operator design limited assessment of long-term outcomes and generalizability. Nevertheless, the prospective design and the combined assessment of symptoms, manometry, and esophageal morphology provide a clinically relevant view of how symptom duration relates to disease phenotype and short-term recovery after POEM.

## Supplementary Information

Below is the link to the electronic supplementary material.

## Data Availability

The datasets generated and/or analyzed during the current study are available from the corresponding author on reasonable request.
