# Supplementary material for: Symptom duration is associated with esophageal remodeling in achalasia treated by peroral endoscopic myotomy
Source: Sci Rep. 2026 Jul 13;16:27537. doi: 10.1038/s41598-026-62349-x (PMC13538632; doi:10.1038/s41598-026-62349-x)
Supplement: Supplementary file 1 [file 41598_2026_62349_MOESM1_ESM.docx]

**Symptom Duration Is Associated With Esophageal Remodeling in Achalasia Treated by Peroral Endoscopic Myotomy**

Chunyu Yang¹, Lanqing Ma¹, Tao Zhang¹, Gang Yang¹, Yuping Wu¹, Rendi Deng¹ and Xiangqian Dong¹*

¹Department of Gastroenterology, The First Affiliated Hospital of Kunming Medical University, Kunming, China

*Corresponding author: Xiangqian Dong, Ph.D., Department of Gastroenterology, The First Affiliated Hospital of Kunming Medical University, Kunming, China. Email: [xiangqian86@163.com](mailto:xiangqian86@163.com)

Supplementary Fig. S1. Step-by-step procedure of POEM. (A) Spastic constriction ring. (B) Submucosal dissection. (C) Creation of the submucosal tunnel. (D) Extension of the submucosal tunnel. (E) Myotomy. (F) Post-myotomy view. (G) Relaxed cardia after myotomy. (H) Closure of the mucosal entry with clips.

**Supplementary Table S1. Details of prior treatment before peroral endoscopic myotomy**

| Patient No. | Symptom-duration group | Symptom duration (months) | Type of prior intervention | Recorded number of prior treatment episodes | Interval from the most recent intervention to POEM |
| --- | --- | --- | --- | --- | --- |
| 4 | 3–10 years | 108 | Endoscopic balloon dilation | 1 | 7 years |
| 8 | >10 years | 156 | Endoscopic balloon dilation | 1 | 4 years |
| 13 | >10 years | 360 | Endoscopic balloon dilation | 1 | 1 year |
| 24 | >10 years | 120 | Esophageal stent placement | 1 | 6 years |
| 25 | <3 years | 12 | Laparoscopic Heller myotomy | 1 | 1 year |
| 30 | >10 years | 156 | Endoscopic balloon dilation | 1 | 10 years |
| 32 | >10 years | 240 | Endoscopic balloon dilation and esophageal stent placement | 1 | 20 years |
| 33 | 3–10 years | 72 | Endoscopic balloon dilation | 1 | 6 years |
| 34 | >10 years | 240 | Endoscopic balloon dilation | 3 | Exact timing unavailable |
| 42 | <3 years | 12 | Prior POEM | 1 | 4 months |
| 49 | >10 years | 240 | Endoscopic balloon dilation | 1 | 20 years |
| 53 | >10 years | 240 | Endoscopic balloon dilation | 1 | 13 years |

The interval denotes the available recorded interval between the most recent prior intervention and the index POEM. For Patient 34, three previous balloon dilations were documented, but the exact timing was unavailable. For Patient 32, balloon dilation and stent placement were recorded together as one prior treatment episode in the source dataset. POEM, peroral endoscopic myotomy.

**Supplementary Table S2. Eckardt score before and after POEM**

| **Group** | **Before POEM** | **3 months** | **P value** | **6 months** | **P value** |
| --- | --- | --- | --- | --- | --- |
| <3 years (n=34) | 7.24 ± 2.20 | 0.88 ± 0.77 | <0.001 | 0.74 ± 0.93 | <0.001 |
| 3-10 years (n=17) | 8.00 ± 2.55 | 1.12 ± 0.93 | <0.001 | 1.05 ± 0.66 | <0.001 |
| >10 years (n=17) | 6.76 ± 2.66 | 1.00 ± 1.12 | <0.001 | 0.88 ± 0.93 | <0.001 |
| Overall (n=68) | 8.00 (6.00-9.00) | 1.00 (0.00-2.00) | <0.001 | 1.00 (0.00-1.25) | <0.001 |

Clinical success was defined as an Eckardt score ≤3

**Supplementary Table S3. Postoperative follow-up outcomes**

| **Variable** | **<3 years (n=34)** | **3-10 years (n=17)** | **>10 years (n=17)** | **P value** |
| --- | --- | --- | --- | --- |
| Eckardt score at 3 months | 1.00 (0.00-1.25) | 1.00 (0.00-2.00) | 1.00 (0.00-2.00) | 0.729 |
| Eckardt score at 6 months | 0.00 (0.00-1.00) | 1.00 (1.00-1.50) | 1.00 (0.00-1.50) | 0.226 |
| Change in Eckardt score, baseline to 3 months | 6.50 (5.00-8.00) | 8.00 (4.50-9.00) | 6.00 (3.50-8.00) | 0.477 |
| Change in Eckardt score, baseline to 6 months | 6.50 ± 2.42 | 6.94 ± 2.49 | 5.88 ± 2.87 | 0.480 |
| Persistent dysphagia at 3 months, n (%) | 11 (32.35) | 10 (58.82) | 9 (52.94) | 0.140 |
| Persistent reflux at 3 months, n (%) | 3 (8.82) | 3 (17.65) | 3 (17.65) | 0.155 |
| Persistent chest pain at 3 months, n (%) | 4 (11.76) | 1 (5.88) | 1 (5.88) | 0.747* |
| Persistent dysphagia at 6 months, n (%) | 10 (29.41) | 6 (35.29) | 6 (35.29) | 0.874 |
| Persistent reflux at 6 months, n (%) | 3 (8.82) | 1 (5.88) | 1 (5.88) | 1.000* |
| Persistent chest pain at 6 months, n (%) | 2 (5.88) | 0 | 0 | 0.493* |
| GerdQ ≥8, n (%) | 3 (8.82) | 1 (5.88) | 4 (23.53) | 0.327* |

* Fisher exact test where applicable

**Supplementary Table S4. Within-group changes in objective parameters after POEM**

| **Parameter** | **<3 years** | **3-10 years** | **>10 years** | **Overall** |
| --- | --- | --- | --- | --- |
| IRP, pre vs post (mmHg) | 26.86 ± 7.07 vs 9.68 ± 5.41; P<0.001 | 24.59 ± 5.91 vs 6.93 ± 0.98; P<0.001 | 14.64 ± 5.92 vs 6.94 ± 1.78; P<0.001 | 23.50 (16.33-30.60) vs 7.80 (5.93-9.40); P<0.001 |
| LES pressure, pre vs post (mmHg) | 34.05 ± 11.44 vs 12.88 ± 8.03; P<0.001 | 34.99 ± 9.93 vs 12.93 ± 5.26; P<0.001 | 16.87 ± 7.96 vs 8.18 ± 2.71; P<0.001 | 29.85 ± 12.72 vs 11.68 ± 6.59; P<0.001 |
| Esophageal dilation, pre vs post (mm) | 39.10 ± 10.89 vs 26.73 ± 10.64; P<0.001 | 47.50 (35.90-58.50) vs 22.80 (20.82-29.00); P<0.001 | 46.00 ± 16.19 vs 32.40 ± 17.94; P<0.001 | 43.33 ± 13.15 vs 27.47 ± 12.19; P<0.001 |
| Distal narrow diameter, pre vs post (mm) | 4.75 ± 1.58 vs 8.13 ± 1.91; P<0.001 | 4.02 ± 0.77 vs 8.18 ± 1.38; P<0.001 | 3.80 (3.40-5.80) vs 8.00 (7.20-9.00); P<0.001 | 4.47 ± 1.36 vs 8.17 ± 1.70; P<0.001 |
| Esophageal angulation, pre vs post (°) | 147.00 (140.00-150.00) vs 148.00 (145.00-150.00); P<0.001 | 133.80 ± 10.21 vs 142.73 ± 5.54; P<0.001 | 125.00 (95.00-141.00) vs 140.00 (115.00-146.00); P<0.001 | 141.50 (126.00-147.25) vs 145.00 (140.00-149.25); P<0.001 |

Postoperative objective follow-up was available for 58 patients

**Supplementary Table S5. Baseline comparison between patients with and without objective 3-month reassessment**

| Variable | Completed objective reassessment (n=58) | No objective reassessment (n=10) | P value |
| --- | --- | --- | --- |
| Age, years | 43.50 (30.75-51.25) | 39.50 (35.75-49.75) | 0.965 |
| Body mass index, kg/m² | 19.49 (17.80-22.00) | 21.35 (18.78-23.83) | 0.171 |
| Symptom duration, years | 3.00 (1.00-10.00) | 1.50 (0.88-9.25) | 0.374 |
| Baseline Eckardt score | 8.00 (6.00-9.00) | 6.00 (3.75-9.00) | 0.225 |
| Median IRP, mmHg | 23.50 (16.33-30.60) | 11.55 (9.28-15.53) | <0.001 |
| LESP, mmHg | 31.35 (18.65-40.98) | 19.80 (11.68-28.45) | 0.014 |
| Esophageal dilation diameter, mm | 41.70 (32.65-52.80) | 38.55 (32.93-48.65) | 0.585 |
| Distal narrow diameter, mm | 4.45 (3.40-5.20) | 4.50 (3.38-6.40) | 0.579 |
| Esophageal angulation, ° | 141.50 (126.00-147.25) | 142.00 (105.50-150.00) | 0.702 |
| Female, n (%) | 30 (51.7) | 3 (30.0) | 0.307* |
| Male, n (%) | 28 (48.3) | 7 (70.0) |  |
| No prior treatment, n (%) | 48 (82.8) | 8 (80.0) | 1.000* |
| Prior treatment, n (%) | 10 (17.2) | 2 (20.0) |  |
| <3 years, n (%) | 28 (48.3) | 6 (60.0) | 0.823* |
| 3–10 years, n (%) | 15 (25.9) | 2 (20.0) |  |
| >10 years, n (%) | 15 (25.9) | 2 (20.0) |  |
| Chicago type I, n (%) | 10 (17.2) | 0 (0.0) | 0.031* |
| Chicago type II, n (%) | 40 (69.0) | 5 (50.0) |  |
| Chicago type III, n (%) | 1 (1.7) | 0 (0.0) |  |
| Unclassified achalasia, n (%) | 7 (12.1) | 5 (50.0) |  |

* Fisher exact test where applicable; for multi-category variables, the P value is displayed on the first category row. IRP integrated relaxation pressure, LESP lower esophageal sphincter pressure

**Supplementary Table S6. Clinical, manometric, and supportive findings in patients with unclassified achalasia**

| ID | Symptom duration, months | Prior treatment | HRM findings | Supportive barium and endoscopic findings |
| --- | --- | --- | --- | --- |
| 8 | 156 | Yes: Endoscopic balloon dilation | IRP 9.2 mmHg; LESP 15.0 mmHg; esophageal body motility did not meet Chicago type I, II, or III criteria. | Distal bird-beak narrowing with mild proximal dilation; tightly closed EGJ with resistance to endoscope passage. |
| 24 | 120 | Yes: Esophageal stent placement | IRP 8.0 mmHg; LESP 13.2 mmHg; esophageal body motility did not meet Chicago type I, II, or III criteria. | Distal bird-beak narrowing with marked proximal dilation and tortuosity; tightly closed EGJ with resistance to endoscope passage. |
| 33 | 72 | Yes: Endoscopic balloon dilation | IRP 8.9 mmHg; LESP 12.8 mmHg; esophageal body motility did not meet Chicago type I, II, or III criteria. | Distal bird-beak narrowing with marked proximal dilation and tortuosity; tightly closed EGJ with resistance to endoscope passage. |
| 36 | 4 | No | IRP 11.7 mmHg; LESP 15.6 mmHg; esophageal body motility did not meet Chicago type I, II, or III criteria. | Distal narrowing with proximal dilation on barium esophagram; tightly closed EGJ with resistance to endoscope passage and a tortuous dilated lumen. |
| 45 | 120 | No | IRP 10.2 mmHg; LESP 16.8 mmHg; esophageal body motility did not meet Chicago type I, II, or III criteria. | Distal bird-beak narrowing with marked proximal dilation and tortuosity; tightly closed EGJ with resistance to endoscope passage. |
| 48 | 360 | No | IRP 7.9 mmHg; LESP 7.2 mmHg; esophageal body motility did not meet Chicago type I, II, or III criteria. | Distal bird-beak narrowing with marked proximal dilation and tortuosity; tightly closed EGJ with resistance to endoscope passage. |
| 49 | 240 | Yes: Endoscopic balloon dilation | IRP 12.1 mmHg; LESP 4.2 mmHg; esophageal body motility did not meet Chicago type I, II, or III criteria. | Distal bird-beak narrowing with marked proximal dilation and tortuosity; tightly closed EGJ with resistance to endoscope passage. |
| 52 | 1 | No | IRP 3.3 mmHg; LESP 14.3 mmHg; esophageal body motility did not meet Chicago type I, II, or III criteria. | Distal bird-beak narrowing with marked proximal dilation; tightly closed EGJ with resistance to endoscope passage. |
| 53 | 240 | Yes: Endoscopic balloon dilation | IRP 12.5 mmHg; LESP 15.4 mmHg; esophageal body motility did not meet Chicago type I, II, or III criteria. | Distal bird-beak narrowing with marked proximal dilation; tightly closed EGJ with resistance to endoscope passage. |
| 56 | 240 | No | IRP 12.3 mmHg; LESP 4.5 mmHg; esophageal body motility did not meet Chicago type I, II, or III criteria. | Distal bird-beak narrowing with proximal dilation; tightly closed EGJ with resistance to endoscope passage. |
| 57 | 24 | No | IRP 9.4 mmHg; LESP 4.9 mmHg; esophageal body motility did not meet Chicago type I, II, or III criteria. | Distal bird-beak narrowing with proximal dilation; tightly closed EGJ with resistance to endoscope passage. |
| 68 | 192 | No | IRP 11.8 mmHg; LESP 8.3 mmHg; esophageal body motility did not meet Chicago type I, II, or III criteria. | Distal bird-beak narrowing with proximal dilation; tightly closed EGJ with resistance to endoscope passage. |

All 12 patients had an IRP <15 mmHg and esophageal body motility that did not meet the classic Chicago type I, II, or III criteria. Five had undergone prior treatment and seven were treatment-naive. They were categorized operationally as unclassified achalasia because the combined clinical, endoscopic, and barium esophagram findings supported achalasia; they were not considered to have an established alternative motility disorder. EGJ esophagogastric junction; HRM high-resolution manometry; IRP integrated relaxation pressure; LESP lower esophageal sphincter pressure

**Supplementary Table S7. Treatment-naive Chicago subtype distribution by symptom-duration group**

| Variable | <3 years (n=32) | 3–10 years (n=15) | >10 years (n=9) | P value |
| --- | --- | --- | --- | --- |
| Type I achalasia | 2 (6.3) | 3 (20.0) | 1 (11.1) | 0.028* |
| Type II achalasia | 26 (81.3) | 12 (80.0) | 4 (44.4) |  |
| Type III achalasia | 1 (3.1) | 0 | 0 |  |
| Unclassified achalasia | 3 (9.4) | 0 | 4 (44.4) |  |

* Fisher exact test across all four subtype categories

**Supplementary Table S8. Treatment-naive Spearman correlations using exact symptom duration in years**

| Analysis | rho | P value | n |
| --- | --- | --- | --- |
| Symptom duration vs IRP | -0.129 | 0.345 | 56 |
| Symptom duration vs LESP | -0.200 | 0.140 | 56 |
| Symptom duration vs esophageal dilation diameter | 0.396 | 0.003 | 56 |
| Symptom duration vs esophageal angulation | -0.572 | <0.001 | 56 |

IRP integrated relaxation pressure, LESP lower esophageal sphincter pressure
